# Impact of COVID-19 on psychological distress in subsequent stages of the pandemic: The role of received social support

**DOI:** 10.1371/journal.pone.0310734

**Published:** 2024-09-25

**Authors:** Krzysztof Kaniasty, Erik van der Meulen

**Affiliations:** 1 Department of Psychology, Indiana University of Pennsylvania, Indiana, PA, United States of America; 2 Institute of Psychology, Polish Academy of Sciences, Warsaw, Poland; 3 Academy of Health and Social Studies, NHL Stenden University of Applied Sciences, Leeuwarden, The Netherlands; St John’s University, UNITED STATES OF AMERICA

## Abstract

This longitudinal study examined a sample of adult Poles (N = 1245), who were interviewed three times from July 2021 to August 2022, during the later stages of the COVID-19 pandemic. The study had two primary objectives. The first was to assess the impact of the pandemic on psychological distress, measured through symptoms of depression and anxiety. The pandemic’s effects were evaluated using three predictors: direct exposure to COVID-19, COVID-19 related stressors, and perceived threats from COVID-19. The second objective was to investigate the role of received social support in coping with the pandemic’s hardships. Receipt of social support was measured by both the quantity of help received and the perceived quality of that support. A Latent Growth Curve Model (LGCM) was employed to analyze psychological distress across three waves, controlling for sociodemographic variables, non-COVID life events, coping self-efficacy, and perceived social support. Findings indicated that COVID-19 stressors and COVID-19 threats were strongly and consistently associated with greater psychological distress throughout the study period. The impact of direct COVID-19 exposure was limited. The quantity of received support predicted higher distress, whereas higher quality of received support was linked to better mental health. Crucially, the relationship between the quantity of support and distress was moderated by the quality of support. Effective social support was associated with the lowest distress levels, regardless of the amount of help received. Conversely, receiving large amounts of low-quality support was detrimental to psychological health. In summary, the ongoing psychosocial challenges of COVID-19 significantly eroded mental health, highlighting the importance of support quality over quantity in coping with significant life adversities.

## Introduction

It is reasonable to assert that the COVID-19 pandemic, like no other collective crisis in the world’s history, prompted an unprecedented number of research studies, reviews and meta-analyses attempting to assess its impact on mental health. Many quantitative and qualitative syntheses documented that the heaviest mental health toll on general public, most frequently assessed as symptoms of depression, anxiety, PTSD, or psychological distress, occurred in the early months of the pandemic [1–4]. Similar patterns of findings emerged within different subgroups, such as COVID-19 patients [5], children and adolescents [6], college students [7], elderly [8] or healthcare workers [9]. Evidence concerning whether in later months of the first year of the pandemic mental health problems decreased [1,4] or remained stable at moderately elevated levels [2,3] is not yet conclusive.

It is also reasonable to assert that the psychological impact of the SARS-CoV-2 virus would persist through subsequent phases of the pandemic. Few, thus far published, longitudinal investigations with mental health assessments conducted after July 2021 [10–15], evidenced overall improvements in psychological health in various populations since the onset of the pandemic. Nevertheless, mental health issues appear elevated as compared to pre-pandemic times [16].

The COVID-19 experience should be regarded as a disaster or catastrophe that set off a prolonged series of diverse and stressful hardships. The pandemic encompassed all possible classes of stressors [17]: traumas (e.g., death, injuries), life events (e.g., lockdowns, job interruptions/loss), daily hassles (e.g., social distancing, mask-wearing), macro-system events (e.g., economic downturns, societal protests/disputes), nonevents (e.g., postponements/cancellations of expected life milestones such as graduations and weddings), and chronic stressors (e.g., ongoing life hardships such as caregiving, environmental challenges). Each of these facets of the COVID-19 catastrophe independently impacted psychological and social well-being, capturing different aspects of the comprehensive spectrum of stress processes [17,18].

The present longitudinal study had two major goals. First, it aimed to assess the impact of the pandemic in its later phases (July 2021—August 2022) on psychological distress assessed as combined symptoms of depression and anxiety. The ongoing presence of the pandemic in people’s lives was measured using three predictor variables. COVID-19 direct exposure for individuals and their significant others was evaluated as probable encounters with the virus. This assessment encompassed a range of experiences from simple testing or mild infection to severe illness, including hospitalization or the death of a significant person. Several COVID-19 studies have documented the association between direct exposure to the SARS-CoV-2 virus and psychological health [19,20]. The second measure, COVID-19 stressors, included a series of significant secondary stressors such as occupational disruptions, financial insecurity, and delays or cancellations. These stressors have also been shown to adversely impact mental health [21,22]. Finally, COVID-19 threats, likely the most frequently assessed indicator of the pandemic’s adversities, evaluated people’s concerns and fears for their own health and the health of their families [21,23].

The second goal of the present study was to investigate the role of social support in the ongoing process of coping with COVID-19 adversities. Social support is a multifaceted construct that encompasses social interactions providing actual assistance and embedding individuals in a network of relationships perceived as loving, caring, and readily available in times of need [24]. The most central distinction between different forms of social support lies between perceived social support and received social support. Perceived social support refers to subjective appraisals of being reliably connected to others, such as believing that "If I needed it, I can easily find someone to talk to about my troubles, worries, or concerns." In contrast, received social support pertains to the actual support received, such as "How often did someone give, loan, or offer you money?"

Perceived social support, regarded as the principal facet of social support, has consistently been shown to be advantageous for better postcrisis outcomes [see 25,26]. Conversely, studies assessing received social support have produced inconsistent findings. Some investigations have documented a clear benefit of greater received support in reducing distress. However, many other studies have found no effects, or worse, have shown positive associations between received support and increased mental health problems [27,28]. Accordingly, the stress and coping literature consistently highlights the benefits of social support for psychological adjustment, with an emphasis on perceived social support rather than received support. This focus poses challenges for public health professionals and practitioners who provide aid, support, and psychological interventions to communities recovering from disasters. It also presents difficulties for countless individuals worldwide who have been striving to offer actual support to one another during the challenging times of the COVID-19 pandemic.

The reasons why the efficacy of received social support may be undermined during times of coping with stressors are extensive [27,29,30]. Providing and receiving help in times of crisis, whether through personal, charitable, or professional relationships, is a complex and challenging process. Good intentions and sincere concerns often mix with confusion, skepticism, and psychological threats. Simply put, while the desire to relieve the suffering of others is commendable, not all forms of social support prove to be helpful.

A number of recommendations can be found in the social support literature that offer ideas for identifying theoretical pathways, along with empirical and practical prerequisites for detecting the genuinely helpful influence of received social support [27,30]. Rini and Dunkel Schetter [31] proposed a comprehensive theoretical framework for investigating the efficacy of received social support, which they labeled the “*social support effectiveness model*” (SSE). The SSE model delineates the joint influence of the “*quantity*” and “*quality*” of received social support and the extent to which helping provisions meet recipients’ expectations, needs and demands from the stressors they face.

The quantity dimension of support receipt is determined by the match between the recipient’s needs and the amount of help received, ensuring the support is neither too little nor too much. The quality dimension involves more complex practical and psychological dynamics, including: a) “functional fit”—the type of help aligns with what is needed; b) “skillfulness and sensitivity”–support is delivered in ways that minimize the recipient’s feelings of being a burden; c) “ease of access”–help is not difficult to get; and d) “impact on self-concept”–the support received does not reflect poorly on one’s self-esteem, avoiding blame, feelings of incompetence, or a sense of indebtedness.

Rini and her colleagues [32] provided strong empirical evidence for the SSE model in a sample of hematopoietic stem cell transplant survivors. When examined together, the quantity of support received was predictive of more distress experienced by survivors, whereas favorable appraisals of the effectiveness of support received were associated with better mental health. Most critically, the two operationalizations of received social support statistically interacted with each other producing a disconcerting pattern revealing that when support was judged as being low in quality, receiving greater quantities of it predicted elevated distress. However, recipients of effective support reported the lowest levels of distress, regardless of the amount of help received. The importance of assessing both the amount and quality of postcrisis received social support for psychological functioning was also evidenced among survivors of disasters [33–35]. Altogether, these findings highlight the importance of enhancing the quality of help provided to people coping with life difficulties. Simply providing "more" support is not necessarily better and can potentially be detrimental if offered in substandard ways. This underscores the need for support that is provided in the right amount and type, delivered with skill and sensitivity, easily accessible, and without negative repercussions for the recipient’s self-image.

In addition to reliance on social support, theory and research on coping with stressful life events repeatedly emphasize the importance of self-efficacy as a critical factor influencing adaptation to significant life challenges, threats, and losses [36,37]. Confidence in one’s own coping abilities and social support resources dynamically influence each other. Received social support may enhance self-efficacy (i.e., enabling path), whereas self-efficacy may mobilize (i.e., cultivation path) social networks to action [38].

The present study examined the role of social support receipt, measured in terms of both quantity and quality, on psychological distress. The analyses accounted for the influence of sociodemographic factors, perceived social support, and beliefs in coping self-efficacy, which are two crucial resources that routinely promote successful coping with stressors. The uniqueness of the COVID-19 catastrophe for studying received social support stems from the fact that everyone has been subjected to its threats, disruptions, and losses. Nearly everyone has needed support at some point, and nearly everyone has provided support at some point.

## Methods

### Sample and procedure

Wave 1 sample was recruited between July 6 and 19, 2021, from an online survey panel (“Ariadna,” a Polish online research panel with over 150,000 registered and verified users) to be representative of Polish adults in terms of gender, age, and size of municipality. It originally consisted of 3074 respondents who met all quality control requirements established for the study based on answers to attention questions, and times of completion of surveys (i.e., participants with completion times faster than 1 *SD* from the sample mean were eliminated). Wave 2 data were collected in February 2022, and Wave 3 followed six months later in August 2022.

The sample analyzed in this study comprised 1,245 respondents who completed all three waves of data assessments and met subsequent (Wave 2 and 3) quality control requirements. A comparison of these participants with those who dropped out after earlier waves of assessments (*N* = 1829, 59.4%) on Wave 1 variables revealed some significant differences. The drop-out participants were younger, less educated, and more likely to live in villages or smaller towns. They were also less likely to be in relationships and had higher scores on the psychological distress measure.

#### Ethics

The study was approved by the Institutional Review Board of the Institute of Psychology, Polish Academy of Sciences (Approvals # Wave 1-13/V/2021, Wave 2-01/1/2022, Wave 3-17/VII/2022). All participants provided written consent prior to each wave of assessments.

### Measures

#### Outcome variable—psychological distress

Symptoms of *psychological distress* were assessed with 8 items from the Patient Health Questionnaire (PHQ-8) [39], and 7 items from the Generalized Anxiety Disorder scale (GAD-7) [40]. These self-reports have been frequently used to assess depressive (e.g., “Little interest or pleasure in doing things”) and anxiety symptoms (e.g., “Feeling nervous, anxious or on edge”). In order to keep our measures consistent across all surveys’ administrations with regards to time frames of responding and response opinions, both instruments asked respondents about how often they were bothered by these symptoms in the last 30 days (instead of the typical for these instruments time frames of “the past two weeks”), with the following five answer choices: 0 (*Never*), 1 (*Rarely*), 2 (*Sometimes*), 3 (*Often*), and 4 (*Very often*). These options were recoded to a four-point scale of the standard PHQ-8 and GAD-7’s response sets (range 0 to 3, with answers “rarely” and “sometimes” both coded as 1). Cronbach’s α reliability coefficients of the PHQ-8 and GAD-7 scores computed as sums were high at all assessment times (0.92–0.94).

The PHQ-8 and GAD-7 are often combined into a single measure of general distress [41], consequently the total score of psychological distress used in the present analyses was a sum of all 15 items. Confirmatory factor analyses using a Diagonally Weighted Least Square Estimator on the present data showed excellent fit for single factor solutions (see S1 Table). Cronbach’s alphas of the psychological distress total scores at each measurement wave were all high (> 0.95).

#### Measurement of focal predictors

*COVID-19 direct exposure* index was based on a sum of answers to 11 questions that asked about exposure to *SARS-CoV-2* in the past 16 (Wave 1) or 6 months (Waves 2 and 3). Questions referred to the participant (e.g., being tested for the virus, if positive how severe was the illness, hospitalization) and to the family and friends (including deaths). Different answer options were used depending on the content of the question, but all responses were recoded as 0 (*No or minimal exposure*) or 1 (*Moderate to severe exposure*).

*COVID-19 stressors* was derived from the average of items that evaluated the extent to which pandemic-specific events (i.e., decline in household budget, irreversible cancellation of important personal events, postponement of important events, new/additional burdens with care for children, new/additional burdens with care of elderly) negatively influenced respondents’ lives in the past 16 months (Wave 1, 10 items) or 6 months (Waves 2 and 3, 6 items; 0 = *Did not happen or not at all*, 4 = *To a great extent*). One additional item was included that asked whether a participant and/or someone in their household experienced COVID-19 related job loss that had negative consequences.

*COVID-19 threats* involved 12 questions asking the participants about their fears and concerns regarding current threats associated with the continuing pandemic (e.g., “I am concerned that someone close to me will get sick with COVID-19, even if it would be a subsequent infection,” “I am worried about difficulties with access to medical personnel with issues not related to COVID-19”). Items were answered using a 7-point Likert-type response option format anchored with 1 (*Definitely disagree*) and 7 (*Definitely agree*). Reliability coefficients of the scores were high at each assessment (>.92).

*Quantity of received social support* was measured by the Inventory of Postdisaster Social Support [42]. Respondents were asked to estimate how often they received different types of help within the timeframe of the past 16 (Wave 1) and 6 months (Waves 2 and 3). For example, a question at Wave 1 asked: “How often, in the last 16 months (i.e., since the beginning of the pandemic), did family members give, loan or offer you money? Regardless of the reason, did this happen…? (1 = *never*, 2 = *rarely*, 3 = *sometimes* 4 = *quite often*, 5 = *very often*). Another example question, from Wave 3 (August 2022), read: How often, in the past 6 months (i.e., from the beginning of February until today), did friends help you understand the situation you were in?

Three types of received support were assessed: emotional (4 items), informational (4 items), and tangible (8 items) support [43]. Each of these 16 items was asked two times to gage amounts of support received from two sources: family/relatives and friends/close acquittances. Thus, the total scale score was an average of 32 items. Reliability coefficients of the scores were high at each assessment wave (>. 96).

*Quality of received social support* was assessed with 12 items modeled on the instrument developed by Rini and Dunkel Schetter [31,32] based on their SSE model. The same six questions, with varying Likert-type five answer options (all coded 1 thru 5), asked respondents for their appraisals of the support received from family/relatives and friends/close acquittances. Respondents judged the help they received along the following dimensions: quantity (“When family members tried to help you, how well did the amount of help you received match the amount of help you wanted?”), functional fit with needs (“How often have you found yourself wishing the help you received had been different—for instance, a different type of help, or offered in a different way or at a different time?), skillfulness of support delivery (“How often did your friends who gave you help provide it skillfully?), ease of getting help (“When you needed help from family members, how often was it difficult to obtain?”; “How often did friends offer you support without you having to ask for it?”), and the overall appraisal of effectiveness of received help (“Broadly speaking, how effective or useful was the help you received from your family?”). Cronbach’s alphas of average scores computed on 12 items were high at each assessment wave (> .85).

#### Measurement of additional predictors

*Normative life events* index was a sum of answers (0 = *No*, 1 = *Yes*) to questions asking whether, in the past 16 (Wave 1) and past 6 months (Waves 2 and 3), respondents experienced any of 19 major life events (e.g., change in marital status, birth of a child/grandchild, other than COVID-19 illness of self or family, not COVID-19 bereavements). The count of non-COVID events was recoded to range from 0 to 9.

*Coping self-efficacy* was measured with six items modeled on the Trauma Self-Efficacy scale [44]. At Wave 1 and 2 the items referred to participants’ perceived capability to cope with challenges and uncertainties of the COVID-19 pandemic (e.g., “Today, how capable are you to successfully deal with your emotions [*anxiety*, *sadness*, *disaffection*, *anger*] related to the pandemic?”; 1 = *Not capable*, 7 = *Very capable*). At Wave 3, the same items were asked about participants’ appraisals of their capability to cope with serious negative life events that might happen to them in the future (e.g., “In the future, when faced with a difficult life circumstance, how capable will you be to successfully deal with emotions [*anxiety*, *sadness*, *disaffection*, *anger*] that you might experience at that time?”). Confirmatory factor analyses with scale items showed acceptable fit for single factor solutions [45]. Internal reliability coefficients of average scores of this scale were high at each wave (> .93).

*Perceived social support* was assessed with 12 items from the Interpersonal Support Evaluation List [46] and 3 items from the Social Provision Scale [47] that asked about an overall perceived availability of emotional (5 items), informational (4 items) and tangible (6 items) social support (e.g., “If I were sick and needed someone to take me to the doctor, I would have no trouble finding that person;” “I have close relationships that provide me with a sense of emotional security and well-being;”1 = *definitely false*; 4 = *definitely true*). Cronbach’s alphas of average scores of this 15-item instrument were high at each wave (> .92).

*Sense of danger due to the war* was also assessed because during the course of this longitudinal research Russia attacked Ukraine (February 24, 2022), a country bordering with Poland. To account for this additional life stressor, participants were asked at Wave 3 (August 2022) to what extent, in the past 30 days, they were afraid, worried, and/or concerned about their own, their family, and the entire country’s safety and welfare due to the ongoing armed conflict (e.g., “To what extent have you felt that life of your family members and relatives were in danger because of the war in Ukraine?”; 1 = *Not at all*, 5 = *To a very great extent; α =* .85) [48].

#### Sociodemographic variables

Five sociodemographic factors were also included in all analyses. Participants’ gender and their marital status were scored as dichotomous variables. Age was scored in years, respondents’ educational attainment was classified into four levels and size of municipality was grouped into five categories.

### Statistical analysis

The lavaan package (version 0.6–9) [49] for R was used to conduct latent growth curve modelling (LGCM) with psychological distress at three waves as an outcome. The latent growth was modelled to be a linear process. Distress was normally distributed (skewness < 0.630 and kurtosis < 0.720 at all three measurement waves) making it feasible to use maximum likelihood estimation for our models.

Three models with increasing complexity were fitted. First, a model with only the psychological distress latent intercept and slope without any predictors was tested. In the next model, the time-invariant predictors of age, gender, educational level, marital status and municipality size were added as predictors of the psychological distress latent intercept and slopes.

**Fig 1:**
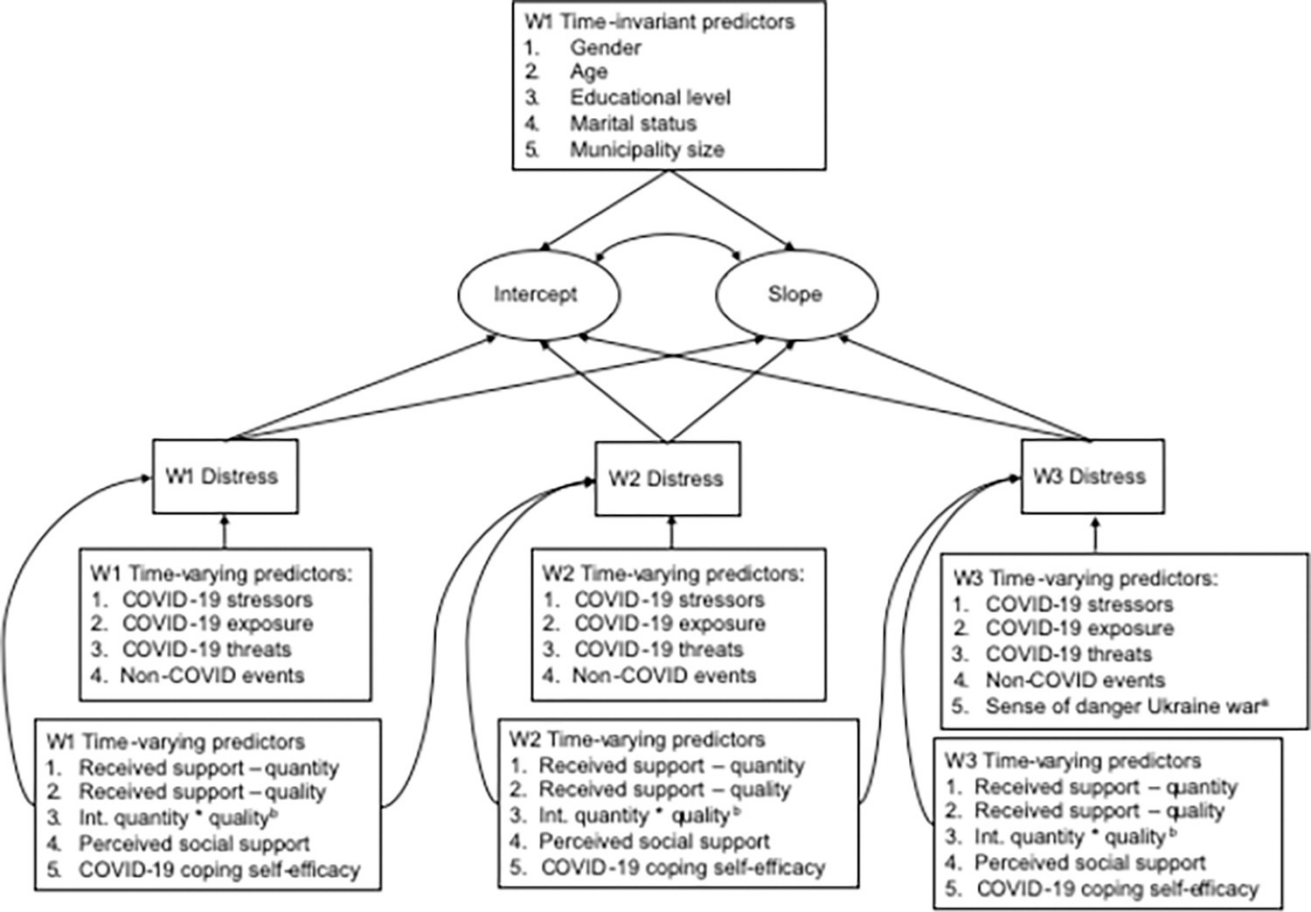
Latent growth curve model with distress as an outcome variable.

The final model of interest in was a model with a latent intercept and slope (using psychological distress measured at three waves), and included time-invariant predictors of the latent intercept and slope (gender, age, educational level, marital status, and municipality size), and time-varying predictors that were measured at all three waves predicting trajectory deviations either only concurrently (COVID-19 exposure, COVID-19 stressors, COVID-19 threat, Non-COVID events), and both concurrently and prospectively (coping self-efficacy, perceived social support, received support-quantity, and received support-quality). Fig 1 gives a full overview of the study model.

A stepwise approach was used to successively fitting models leading up to more complex models, running from a growth curve model only to the addition of both time-invariant and time-varying predictors. First, we fitted a model with only the growth curve, which included a latent intercept and slope. Next, we enhanced the model by adding the time-invariant predictor. Finally, we further refined the model by incorporating the time-varying predictors. All variables were mean-centered before being entered into the conditional models. The following model fit statistics were used: *χ*^*2*^ (and its significance), RMSEA (and its confidence interval), CFI, NFI and SRMR. Using Hu and Bentler’s [50] criteria, a CFI and NFI close to .95, an SRMR close to .06 and an RMSEA close to 0.08 were indications of adequate fit.

Post-hoc analyses on the interaction effects were conducted by categorizing the quality of received support into three levels (< - 1 *SD*, -1 *SD* to + 1 *SD*, > + 1 *SD*). Subsequently, a simple regression of predicted distress scores (retrieved from the most complex LGCM) on the quantity of received support for each category were conducted.

## Results

Table 1 provides an overview of descriptive statistics and S2 Table provides correlations for all variables (*N* = 1047; participants who reported receiving no support at any of three measurement times were excluded).

**Table 1:** Sample descriptive statistics (*N* = 1245).

		Wave 1	Wave 2	Wave 3
		July 2021	February 2022	August 2022
		M (Sd)/n (%)	M (Sd)/n (%)	M (Sd)/n (%)
Gender				
	Women	695 (55.8)		
	Men	550 (44.2)		
Age		51.9 (14.2)		
Educational level				
	< High school	104 (8.4)		
	High school	398 (32.0)		
	Post high school	151 (12.1)		
	BA, MA and higher	592 (47.6)		
Marital status				
	Unmarried	377 (30.3)		
	Married	868 (69.7)		
Municipality size				
	Village	356 (28.6)		
	Town	140 (11.2)		
	Medium-sized city	263 (21.1)		
	Large city (100–500k)	277 (22.2)		
	Large city (> 500k)	209 (16.8)		
Psychological distress		14.63 (8.48)	14.91 (8.71)	14.54 (8.55)
COVID-19 exposure		2.81 (1.96)	1.96 (1.58)	1.00 (1.39)
COVID-19 stressors		0.54 (0.46)	0.43 (0.59)	0.28 (0.46)
COVID-19 threats		5.04 (1.28)	4.94 (1.35)	4.21 (1.52)
Received support-quantity		1.98 (0.62)	2.10 (0.67)	2.02 (0.70)
Received support-quality		3.50^a^ (0.78)	3.53^b^ (0.76)	3.51^c^ (0.76)
Perceived social support		3.12 (0.57)	3.09 (0.56)	3.08 (0.56)
COVID-19 coping self-efficacy		5.28 (1.23)	5.29 (1.16)	4.68 (1.15)

^a^ N = 1220

^b^ N = 1145

^c^ N = 1092.

### Model fit

In total three models were tested (unconditional model, conditional model with only time-invariant predictors and conditional model with time-invariant and time-varying covariates). Before we modelled our intended model, we assessed: 1) potential multicollinearity among predictors and 2) potential overfit of the model (given the number of predictors). Multicollinearity was assessed by examining correlations among the predictor variables. Of the 528 correlations possible among all predictors, 24 were larger than 0.5 or smaller than -0.5 (4.5%).

These stronger correlations existed among the same variables measured at different times and between COVID-19 coping self-efficacy and psychological distress. To determine whether these correlations raised multicollinearity issues in the LGCM, three multiple regression models were run with the predicted distress scores at each wave as dependent variables and the LGCM-corresponding time-varying covariates as independent variables. Independent variable’s variance inflation factors (VIFs) of these models never exceeded values of 2.871 which was well under the threshold of 5 and, thus, signaling no obvious multicollinearity problems.

Overfit of the model was assessed by changes in the Akaike’s Information Criterion (AIC) of the predictors in relation to a model without predictors—a decrease of the AIC was indicative of an enhanced model fit when the particular predictor was added to the model. We examined both the bivariate decreases in AIC for each predictor (i.e. differences in AIC between every predictor separately to a model without any predictors) and hierarchical decreases in AIC (i.e. successively adding predictors and determining the decrease in AIC after each addition). Some variables appeared to add little to the model and caused a slight increase in the AIC. However, these decreases were relatively small and their negative impact on model fit, thereby, was rather minor. For reasons of completeness, these variables were kept in the model, nonetheless. S3 Table gives a full overview of overfit assessment. An additional consideration for overfit is the adequacy of the sample size in relation to model complexity. This can be captured by the ratio of estimated parameters to the number of respondents [51,52]; a minimum is 1 to 5 (i.e. 5 respondents for every estimated parameter), for the current study this was 1 to 18.70 highlighting an exceedingly sufficient sample size. Therefore, our modelling approaches were deemed valid.

### Latent growth

All models, one unconditional and two conditional models, yielded significant latent intercepts and non-significant latent slopes. The first rows of Table 2 indicate the latent growth factors (intercept and slope) for each model. The non-significant slopes in all three models reflect a general absence of change over time. Only in the unconditional model, the latent intercept and slope were associated; individuals with higher initial starting values showed a higher decline over time (*cov* = .177). In both conditional models, the latent intercept and slope were unrelated (*cov* = .113 and .001, respectively).

**Table 2:** Outcomes of distress latent growth curve models (N = 1047).

			Model 1	Model 2	Model 3
			*β* (SE)	*β* (SE)	*β* (SE)
Growth model					
	Intercept		1.964 (0.069)***	1.982 (0.069)***	2.961 (0.128)***
	Slope		-0.040 (0.052)	-0.051 (0.058)	-0.232 (0.217)
	Cov. intercept and slope		-0.177 (0.077)*	-0.113 (0.096)	0.001 (0.312)
Time-invariant predictors of latent intercept					
	Gender			-0.173 (0.033)***	-0.139 (0.038)***
	Age			-0.173 (0.033)***	-0.201 (0.039)***
	Educational level			-0.009 (0.033)	0.001 (0.038)
	Marital status			-0.012 (0.033)	0.004 (0.038)
	Municipality size			-0.017 (0.034)	-0.030 (0.039)
Time-invariant predictors of latent slope					
	Gender			0.099 (0.060)	0.369 (0.303)
	Age			0.103 (0.062)	-0.034 (0.128)
	Educational level			-0.010 (0.057)	0.042 (0.121)
	Marital status			0.028 (0.057)	0.163 (0.171)
	Municipality size			0.051 (0.058)	0.185 (0.183)
W1 Time-varying predictors-concurrent					
	W1 COVID-19 exposure				0.003 (0.020)
	W1 COVID-19 stressors				0.066 (0.022)**
	W1 COVID-19 threat				0.156 (0.022)***
	W1 Non-COVID events				0.053 (0.020)**
	W1 Received support-quantity				0.150 (0.025)***
	W1 Received support-quality				-0.178 (0.026)***
	W1 Interaction quantity X quality				-0.075 (0.021)***
	W1 Perceived support				-0.131 (0.026)***
	W1 COVID-19 coping self-efficacy				-0.311 (0.023)***
W2 Time-varying predictors-concurrent					
	W2 COVID-19 exposure				0.060 (0.018)**
	W2 COVID-19 stressors				0.072 (0.020)***
	W2 COVID-19 threat				0.123 (0.021)***
	W2 Non-COVID events				0.019 (0.018)
	W2 Received support-quantity				0.096 (0.026)***
	W2 Received support-quality				-0.093 (0.026)***
	W2 Interaction quantity X quality				0.034 (0.021)
	W2 Perceived support				-0.117 (0.029)***
	W2 COVID-19 coping self-efficacy				-0.285 (0.025)***
W2 Time-varying predictors-prospective					
	W1 Received support-quantity				0.089 (0.026)**
	W1 Received support-quality				-0.069 (0.027)*
	W1 Interaction quantity X quality				-0.029 (0.020)
	W1 Perceived support				-0.012 (0.028)
	W1 COVID-19 coping self-efficacy				-0.130 (0.025)***
W3 Time-varying predictors-concurrent					
	W3 COVID-19 exposure				0.018 (0.019)
	W3 COVID-19 stressors				0.040 (0.021)
	W3 COVID-19 threats				0.132 (0.023)***
	W3 Non-COVID events				0.124 (0.019)***
	W3 Sense of danger Ukraine war				0.059 (0.022)**
	W3 Received support-quantity				0.134 (0.028)***
	W3 Received support-quality				-0.120 (0.028)***
	W3 Interaction quantity X quality				-0.020 (0.022)
	W3 Perceived support				-0.076 (0.031)*
	W3 COVID-19 CSE				-0.117 (0.021)***
W3 Time-varying predictors-prospective					
	W2 Received support-quantity.				0.025 (0.029)
	W2 Received support-quality.				-0.052 (0.030)
	W2 Interaction quantity X quality				0.020 (0.024)
	W2 Perceived support				-0.058 (0.032)
	W2 COVID-19 coping self-efficacy				-0.224 (0.026***
Residual variance					
	Intercept		NA	0.934 (0.016)***	0.933 (0.019***
	Slope		NA	0.972 (0.022)***	0.794 (0.330)*
	W1 Distress		0.177 (0.036)***	0.193 (0.034)***	0.266 (0.029***
	W2 Distress		0.245 (0.016)***	0.240 (0.016)***	0.216 (0.014***
	W3 Distress		0.140 (0.036)***	0.151 (0.035)***	0.218 (0.029)***
Model fit					
	χ2 (*df*)		4.95 (1)	13.19 (6)	229.44 (52)
	χ2 *p*-value		.026	.040	.000
	CFI		.998	.997	.943
	NFI		.998	.994	.928
	RMSEA		.061	.034	.057
	95% CI RMSEA		[.061, .120]	[.034, .059]	[.057, .065]
	SRMR		.011	.008	.023

*Note*. *β* = standardized parameter estimate; SE = standard error; W1 = Wave 1; W2 = Wave 2; W3 = Wave 3; cov = covariance; CFI = comparative fit index; NFI = normative fit index; RMSEA = root mean square error of approximation; SRMR = standardized root mean square residual. ^a^ Women were the reference category.

* p < .05

** p < .01

*** p < .001.

#### Time-invariant predictors

In the model with only time-invariant predictors (Model 2; see Table 2), the latent intercept was associated with gender and age; women and younger respondents were more distressed initially. None of the time-invariant predictors were predictive of the latent slope.

#### Time-varying predictors: COVID-19 variables, non-COVID life events, and sense of danger due to the war

The last column of Table 2 (Model 3) conveys the outcomes of time-varying predictors. The COVID-19 experiences variables (COVID-19 exposure, stressors and threats) and the experience of non-COVID events were assessed as concurrent predictors (i.e. *i*^th^ wave to *i*^th^ wave) of distress at each wave. Of these variables, the COVID-19 stressors and COVID-19 threats, and non-COVID events were significantly associated with distress at each wave. Higher levels of stressors, threats and other life events were associated with more symptoms of distress. COVID-19 exposure was only significantly positively associated with distress at Wave 2; i.e., more virus exposure was predictive of with more distress. Sense of danger due to the Russian-Ukrainian war significantly predicted higher levels of symptom at Wave 3.

#### Time-varying predictors: Coping self-efficacy, perceived social support, quantity and quality of received social support

Coping self-efficacy ratings were strongly both concurrently and prospectively (i.e. *i*^th^ wave to *i+1*^th^ wave) associated with lower distress scores at all waves. Perceived social support was concurrently associated with lower levels of distress symptoms, but never prospectively.

Quantity and quality of received support were concurrently associated with distress at all three measurement moments. Prospectively, both Wave 1 quantity and quality of received support were predictive of later distress only at Wave 2. Received support quantity was positively associated with psychological distress, such that greater amounts of support were associated with more distress. However, appraisals of the quality of received support were negatively associated with distress, such that greater quality of received support was associated with lower levels of distress symptoms.

**Fig 2:**
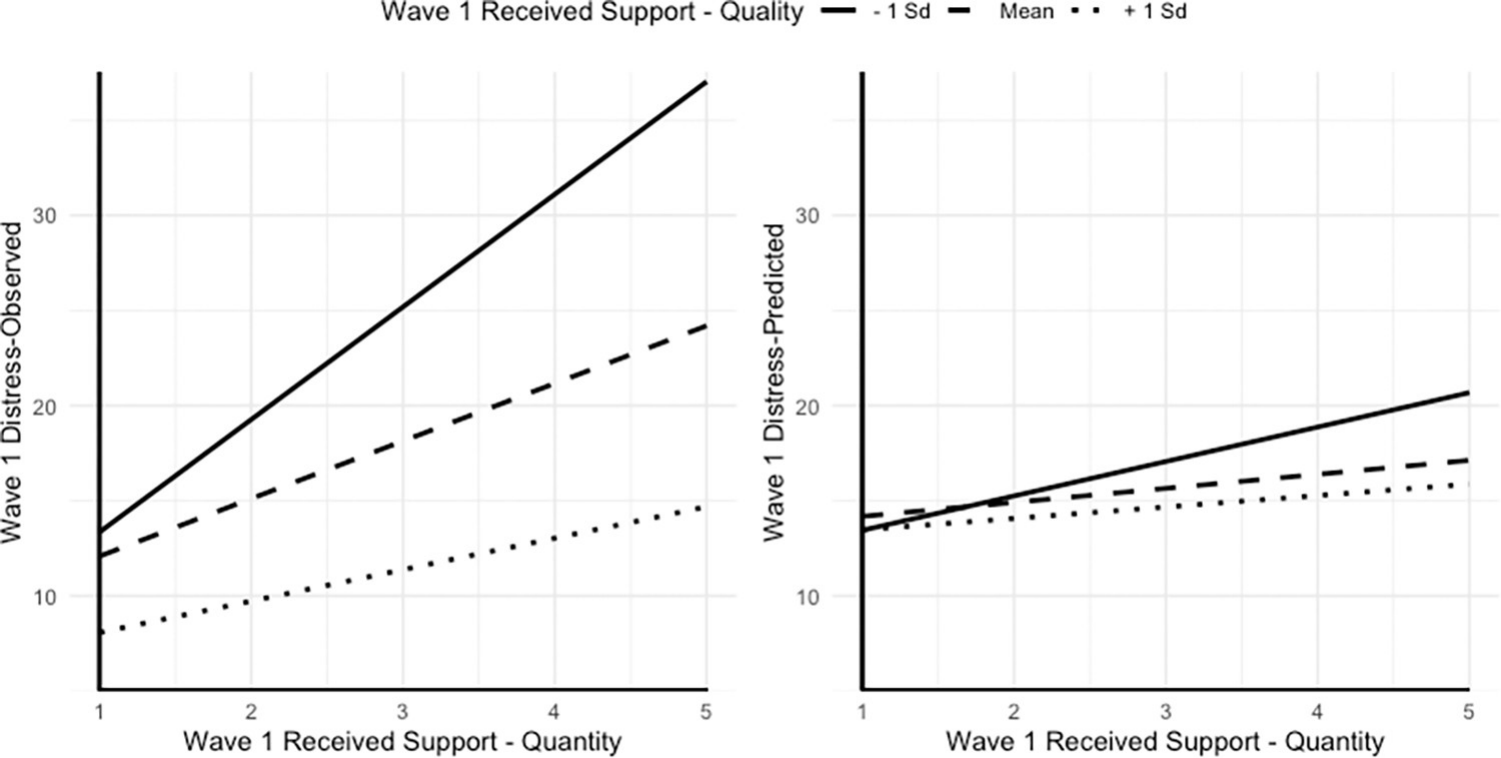
Interaction Effect of Received Support Quantity with Quality on Observed (left pane) and Predicted Distress Scores (right pane). Predicted scores were retrieved from the Latent Growth Curve Model including all time-invariant and time-varying predictors.

The interaction between Wave 1 quantity of received support by Wave 1 quality of received support was statistically significant predicting Wave 1 distress. Fig 2 presents the plots of this interaction associated with observed (left panel) and predicted distress scores (right panel). Persons who judged support received as low in quality reported the highest levels of distress, and greater amounts of received help were strongly associated with higher levels of distress (post-hoc slope analyses, *B* = 1.810, *p* = .030). The slope for the average quality of received support group was also statistically significant (*B* = 0.737, *p* = .020) but the adverse effect of the amount of received support was less pronounced. Most importantly, however, persons who received most efficacious support reported lowest levels of symptoms compared to the other two groups, and the amount of help they actually received did not influence of their experience of distress *(B* = 0.607, *p* = .207). No other quantity by quality interactions were statistically significant.

## Discussion

Fundamentally, the experience of COVID-19 could be considered a total catastrophic event because the pandemic spurred all possible classes of stressors [17]. It has been a traumatic and/or major life changing event, it created daily hassles, it caused macro-system turbulences, generated a surplus of disappointing nonevents, and many of its repercussions have evolved into identifiable chronic stressors. All these facets of the COVID-19 pandemic represent separate parts of the overall universe of stress processes, each potentially adversely influencing mental health.

The present study examined psychological distress trajectories a sample of adult Poles who were interviewed three times from July 2021 to August 2022, thus during later stages of the pandemic. A Latent Growth Curve Model (LGCM) revealed that respondents differed in their level of psychological distress, although changes in these trajectories were generally absent. In other words, individual growth trajectories only differed in the level of distress, but all trajectories were horizontal. Relative stability of the pandemic-related symptomatology was also documented in the meta-analysis of prevalences of depression reported by studies conducted during the first year of the pandemic [2]. Similarly to prior COVID-19 studies, the levels of mental health were dependent on gender and age with women and younger respondents exhibiting more symptoms [2,3,6,7,9].

COVID-19 stressors and COVID-19 threats were both strongly and consistently associated with greater distress throughout the study. The influence of COVID-19 direct exposure was limited to one assessment period. Notwithstanding the overall traumatic and grave consequences of the SARS-CoV-2 virus, it can be said that the pandemic’s psychosocial challenges and disturbances have most forcefully eroded mental health [21,22]. Continuing effect of COVID-19 pandemic on distress in the present sample was observed controlling for harmful influences of other normative life events and sense of danger associated with Russia’s invasion of Ukraine [48,53].

There are many psychological and social resources that empower humans to show resilience and recover successfully from adversity. Chief among them are survivors’ sense of trust in their own ability to face demands/losses posed by the stressor [see 36,37,54] and perceptions of being supported [see 55,56]. In accord with other investigations of the pandemic, results of the present study showed that higher levels of coping self-efficacy [57–59] and perceived social support [60–63] were consistently associated with lower levels of distress symptomatology.

The main interest of this research was focused on mental health influence of the amount of received social support and appraisals of its quality. The few available COVID-19 studies that investigated the quantity of actual receipt of help have produced mixed findings, yielding very limited beneficial effects [64,65], or no effects at all [59,66]. Contradictory evidence was also reported suggesting that the amount of received support was associated with lower distress [67,68], or with greater distress [69]. On the one hand, the results of the present analyses showing adverse psychological effects of receiving greater levels of support could just add to this confusion. However, more favorable appraisals of effectiveness of received support showed a protective function and, with equal consistency, were associated with lower levels of psychological distress. The pattern of the received support quantity by its quality interaction offers a reasonable and theory-based (SSE model) [31] interpretation of this apparent inconsistency. Persons who received effective social support exhibited the lowest levels of distress symptoms, irrespective of the amount of help. On the other hand, receiving large amounts of ineffective social support appeared to be detrimental to mental health. These results replicated an interaction pattern reported by Rini et al.’s [32] and should warn potential social support providers that *if they cannot help smart*, *they should not attempt to help that hard*. In other words, as long as it is delivered in an efficacious manner, received social support protects mental health in the context of stressful circumstances [33–35].

### Strengths and limitations

The use of LGCM allowed to model psychological distress trajectories and predicting distress trajectory deviations from factors that were both stable and changed over time. In other words, the model depicted individuals’ typical distress trajectories and identified why and when individual’s had a-typical distress levels influenced by a comprehensive set of (possible) experiences along the trajectory, most notably: COVID-19 experiences and received support. Conservative analyses included, as control factors, relevant sociodemographic variables, potentially stressful life events not related to the pandemic, and participants’ concerns about the ongoing war in neighboring Ukraine. The study’s sample was large and randomly selected from a nationally representative internet panel. However, across the study’s three assessments, close to 60% of the initial sample was not retained due to attrition and strict data quality control procedures. In addition, all typical disadvantages associated with longitudinal online surveys apply. Finally, although the quantity by quality of received support interaction was consistent with theoretical underpinnings of the study it reached statistical reliability only one time. Thus, this interactive effect should be viewed with prudence as it requires additional examinations.

## Conclusion

Although the rates of severe illness and deaths due to infections with variants of the coronavirus SARS-CoV-2 have gradually decreased and vaccination campaigns continue to reach more and more people, it is not unreasonable to assert that adverse mental health impact of the COVID-19 pandemic will persist. Results of the present study suggest that the ongoing presence of COVID-19 concerns, disturbances and losses have become chronic stressors. Citizens of the world may have to “domesticate” these challenges along with mastering personal and collective strategies to prevent and mitigate harmful psychological consequences of the pandemic. Clearly, beliefs in coping self-efficacy and sense of being reliably connected to others serve as robust contributors to successful coping and adaptation. The conditions under which actually receiving social support are less straightforward, particularly in the context of community-wide emergencies that routinely call for considerable amounts of help and assistance. What appears decisive when aiding people in times of coping with a variety of stressors is the quality, not necessarily quantity, of support provided. In our private as well as professional roles as helpers, it is worth remembering that the benefits of support provided to others may be achieved more readily if we attempt to *help smarter rather than harder*.

## Supporting information

S1 Table Confirmatory analysis of single factor distress scale composed of GAD-7 and PHQ-8. (PDF)

S2 Table Correlations among study variables (n = 1047). (XLSX)

S3 Table Assessment of model overfit and incremental value of predictors. (PDF)
