# Supplementary material for: Impact of COVID-19 on psychological distress in subsequent stages of the pandemic: The role of received social support
Source: PLoS One. 2024 Sep 25;19(9):e0310734. doi: 10.1371/journal.pone.0310734 (PMC11423997; doi:10.1371/journal.pone.0310734)
Supplement: S1 Table — (PDF) [file pone.0310734.s001.pdf]

**S1 Table 1. Confirmatory analysis of single factor distress scale composed of GAD-7 and PHQ-8**

| | Wave 1<br>Est. (SE) | Wave 2<br>Est. (SE) | Wave 3<br>Est. (SE) |
|---|---|---|---|
| GAD 1 | 0.806 (0.018) | 0.786 (0.019) | 0.813 (0.019) |
| GAD 2 | 0.782 (0.019) | 0.788 (0.020) | 0.772 (0.020) |
| GAD 3 | 0.740 (0.016) | 0.776 (0.017) | 0.726 (0.016) |
| GAD 4 | 0.806 (0.016) | 0.814 (0.016) | 0.801 (0.016) |
| GAD 5 | 0.751 (0.016) | 0.728 (0.016) | 0.707 (0.016) |
| GAD 6 | 0.792 (0.016) | 0.783 (0.017) | 0.798 (0.016) |
| GAD 7 | 0.830 (0.016) | 0.835 (0.016) | 0.825 (0.016) |
| PHQ 1 | 0.701 (0.017) | 0.735 (0.017) | 0.705 (0.016) |
| PHQ 2 | 0.854 (0.017) | 0.862 (0.017) | 0.856 (0.018) |
| PHQ 3 | 0.705 (0.016) | 0.699 (0.016) | 0.702 (0.016) |
| PHQ 4 | 0.720 (0.021) | 0.749 (0.019) | 0.732 (0.020) |
| PHQ 5 | 0.713 (0.015) | 0.717 (0.015) | 0.713 (0.015) |
| PHQ 6 | 0.780 (0.015) | 0.781 (0.015) | 0.787 (0.016) |
| PHQ 7 | 0.776 (0.016) | 0.808 (0.017) | 0.777 (0.016) |
| PHQ 8 | 0.641 (0.015) | 0.646 (0.016) | 0.620 (0.016) |
| CFI | 0.997 | 0.998 | 0.997 |
| RMSEA | 0.035 | 0.031 | 0.033 |
| 95%CI RMSEA | [0.029, 0.042] | [0.025, 0.038] | [0.026, 0.039] |
| SRMR | 0.041 | 0.039 | 0.040 |

*Note.* Estimation was based on Diagonally Weighted Least Squares; All estimates were significant at $p<.001$; GAD = Generalized Anxiety Disorder Scale; PHQ = Patient Health Questionnaire; CFI = comparative fit index; RMSEA = root mean squared error of approximation; SRMR = standardized mean squared residual.
