# Supplementary material for: Impact of COVID-19 on psychological distress in subsequent stages of the pandemic: The role of received social support
Source: PLoS One. 2024 Sep 25;19(9):e0310734. doi: 10.1371/journal.pone.0310734 (PMC11423997; doi:10.1371/journal.pone.0310734)
Supplement: S3 Table — (PDF) [file pone.0310734.s003.pdf]

Supplementary Table 3.

Assessment of Model Overfit and Incremental Value of Predictors

	Bivariate[a]		Hierarchical[b]	
	AIC	ΔAIC	AIC	ΔAIC
Empty model	20192.90		20192.90	-
Time-invariant				
Gender	20166.24	-26.66	20166.24	-26.66
Age	20165.89	-27.01	20142.55	-23.69
Education	20196.86	3.96	20146.42	3.87
Marital status	20196.03	3.13	20150.18	3.76
Municipality size	20194.08	1.18	20153.35	3.17
Time-varying concurrent				
COVID-19 exposure	20188.08	-4.82	20147.82	-5.53
COVID-19 stressors	20112.47	-80.43	20072.69	-75.13
COVID-19 threats	20074.83	-118.07	19972.15	-100.54
Non-COVID events	20144.08	-48.82	19929.79	-42.36
Sense of danger due war	20178.88	-14.02	19920.49	-9.30
Received-quantity	20171.78	-21.12	19915.56	-4.93
Received-quality	20117.17	-75.73	19803.86	-111.70
Interaction quant X quali	20187.86	-5.04	19798.61	-5.25
Perceived support	20136.57	-56.33	19728.78	-69.83
COVID-19 self-efficacy	19941.82	-251.08	19506.54	-222.24
Time-varying prospective				
Received-quantity	20192.01	-0.89	19505.73	-0.81
Received-quality	20185.11	-7.79	19489.07	-16.66
Interaction quant X quali	20194.25	1.35	19491.01	1.94
Perceived support	20194.35	1.45	19486.99	-4.02
COVID-19 self-efficacy	20154.28	-38.62	19385.63	-101.36

Note. AIC = Akaike's Information Criterion.

[a] AIC differences of individual predictors compared to the empty model (i.e. row one).

[b] AIC differences of predictors were assessed incrementally after each addition of a predictor to the model (AIC differences = i^{th} row versus i-1th row).
